# The Pro-Oxidant Activity of Pheomelanin is Significantly Enhanced by UVA Irradiation: Benzothiazole Moieties Are More Reactive than Benzothiazine Moieties

**DOI:** 10.3390/ijms19102889

**Published:** 2018-09-23

**Authors:** Hitomi Tanaka, Yui Yamashita, Kana Umezawa, Tomohisa Hirobe, Shosuke Ito, Kazumasa Wakamatsu

**Affiliations:** 1 Department of Chemistry, Fujita Health University School of Health Sciences, 1-98 Dengakugakubo, Kutsukake-cho, Toyoake, Aichi 470-1192, Japan; hitanaka@u-gifu-ms.ac.jp (H.T.); im-y-pi.05@gmail.com (Y.Y.); umezawakana914@gmail.com (K.U.); sito@fujita-hu.ac.jp (S.I.); 2 Laboratory for Cell Culture and Pathology, Shinjuku Skin Clinic, Kawase Building BF1, Shinjuku 3-17-5, Shinjuku-ku, Tokyo 160-0022, Japan; hirobe.tomohisa@qst.go.jp

**Keywords:** pheomelanin, mouse, benzothiazole, benzothiazine, pro-oxidant activity, melanoma, reactive oxygen species, ultraviolet-A, glutathione

## Abstract

It is generally considered that eumelanin (EM) is photoprotective while pheomelanin (PM) is phototoxic. A recent study using a mouse model demonstrated that PM produces reactive oxygen species (ROS) that cause DNA damage and eventually lead to melanomagenesis. A biochemical study showed that PM possesses a pro-oxidant activity. PM consists of benzothiazine (BT) and benzothiazole (BZ) moieties, BT moieties being transformed to BZ moieties by heat or light. In this study, we compared the effects of ultraviolet A (UVA) irradiation using synthetic PMs with different BT to BZ ratios and using various coat color mouse hairs. We found that UVA irradiation of BZ-PM increased glutathione (GSH) depletion and generated more H_2_O_2_ than UVA irradiation of BT-PM. Non-irradiated controls did not exhibit strong pro-oxidant activities. Upon UVA irradiation, yellow mouse hairs oxidized GSH and produced H_2_O_2_ faster than black or albino mouse hairs. Next, to examine the mechanism of the pro-oxidant activity of BT-PM and BZ-PM, we examined the pro-oxidant activities of 7-(2-amino-2-carboxyethyl)-dihydro-1,4-benzothiazine-3-carboxylic acid (DHBTCA) and 6-(2-amino-2-carboxyethyl)-4-hydroxybenzothiazole (BZ-AA) as BT and BZ monomers, respectively. Their pro-oxidant activities were similar, but a large difference was seen in the effects of ROS scavengers, which suggests that the redox reactions may proceed via singlet oxygen in BZ-AA and via superoxide anions in DHBTCA. These results show that UVA enhances the pro-oxidant activity of PM, in particular BZ-PM.

## 1. Introduction

Melanoma is a malignant skin tumor that arises from melanocytes. The incidence of melanoma is increasing worldwide every year, especially in subjects with fair skin and light-colored hair [1]. Melanin pigments of mammals and birds are produced within melanosomes in melanocytes and consist of black to brown eumelanin (EM) and yellow to reddish-brown pheomelanin (PM) [2,3,4]. It is generally accepted that EM is photoprotective, while PM is phototoxic to pigmented tissues [5,6,7,8]. For example, a recent study by Fajuyigbe et al. examined the DNA protection factors (DNA-PFs) of constitutive melanin by comparing fair skin with dark skin and showed that the DNA-PF of melanin is dependent on its epidermal localization and comparable to the differences in keratinocyte carcinoma incidence between light and black skin types [9]. However, recent studies reported that even EM can also be phototoxic [10,11,12]. The ultraviolet (UV) portion of sunlight that reaches the earth’s surface is comprised of UVA (ultraviolet A) (320–400 nm) and UVB (290–320 nm). Both UVA and UVB lead to tanning of human skin following sun exposure. PM has only a weak protective capacity against UV radiation relative to EM, but conversely PM is highly phototoxic and can amplify the UV-induced production of reactive oxygen species (ROS) that cause DNA damage [13,14,15,16]. UVB directly damages DNA, which leads to the transcriptional activation of melanogenic enzymes, resulting in delayed tanning [17,18,19]. In contrast, UVA causes oxidative changes in melanin and leads to immediate pigment darkening and eventually to persistent pigment darkening [10]. Thus, UVB promotes pigmentation that is photoprotective, whereas UVA induces photooxidation of preexisting melanin pigments that may not be photoprotective [17,18].

In addition to UVB, UVA is now considered to be carcinogenic [20], but its exact role in the induction of melanoma is yet to be clarified. In this relation, it should be mentioned that Noonan et al. [21] reported that melanoma induction by UVA requires the presence of melanin pigment and is associated with oxidative DNA damage within melanocytes. That study irradiated neonatal C57BL/6-HGF transgenic black mice with UVA and compared its effects with effects on C57BL/6-c HGF transgenic albino mice. A recent study by Lawrence et al. has also shown that biologically significant DNA damage occurred even at UV/visible boundary wavelengths (385–405 nm) in vitro in keratinocytes and in vivo in human volunteers [22].

**Figure 1:**
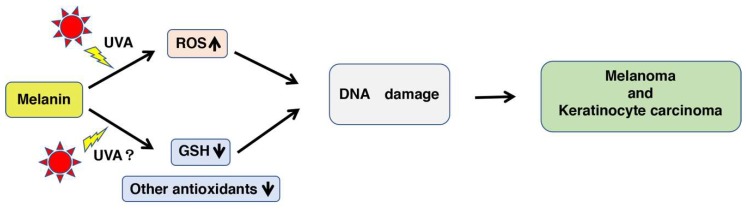
The increased production of ROS (reactive oxygen species) and the decreased antioxidative activities by UVA (ultraviolet A) irradiation of melanin causes DNA damage that leads to melanomagenesis. Melanin pigments, especially pheomelanin (PM), are sensitive to UVA, giving rise to ROS that directly damage DNA, leading to an increased risk for melanoma and keratinocyte carcinoma [9]. Although there is no direct evidence for the relative abundance of PM in fair skin [35], it is known that melanocytes from fairer-colored eyes contain more PM-rich melanin than those from darker-colored eyes [36]. On the other hand, PM oxidizes glutathione (GSH) and other antioxidants. This pro-oxidant activity could be enhanced by UVA radiation, and the decrease of antioxidants indirectly damages DNA. Yellow arrow indicates the irradiation of UVA. “UVA?” shows whether UVA irradiation of melanin decreases GSH or not is unknown well.

The formation of PM is biochemically sustained by elevated levels of cysteine (CySH) in melanosomes, initiated by the production of 5-*S*-cysteinyldopa (5*S*CD) and minor amounts of 2-*S*-cysteinyldopa (2*S*CD). The oxidation of cysteinyldopa isomers leads to benzothiazine (BT) intermediates that undergo dimerization, cross-coupling, and/or ring contraction to benzothiazole (BZ) moieties through complex reactions [23,24,25,26,27]. In 1978, Chedekel et al. reported that photoexcited PM generates superoxide anions, a finding that first suggested a photosensitization mechanism as the key to UV-induced cell damage in red-haired individuals [14] (Figure 1). In addition to the long recognized UV-dependent pathway of toxicity and cell damage, UV-independent melanoma carcinogenesis induced by PM has recently been disclosed using mice with a red hair/fair skin phenotype [28]. Redox properties have been described for synthetic PMs and mouse hairs having a phenotype analogous to red hair/fair skin humans [28,29]. Furthermore, pheomelanosomes are more pro-oxidant than eumelanosomes [30] and synthetic PM produces superoxide anions even in the absence of UV radiation [31]. The concept of melanin being a redox buffer has been known for years. However, a recent study by Kim et al. reinforced that concept by applying an electrochemically based reverse engineering methodology to compare the redox properties of PM and EM [32]. Their results demonstrated that both PM and EM are redox-active, and that PM possesses a greater oxidative redox potential than EM. Thus, the UV-independent production of superoxide anions and the depletion of cellular antioxidants by PM (pro-oxidant activity) opens a new avenue of research in melanin chemistry and biochemistry [33,34].

**Figure 2:**
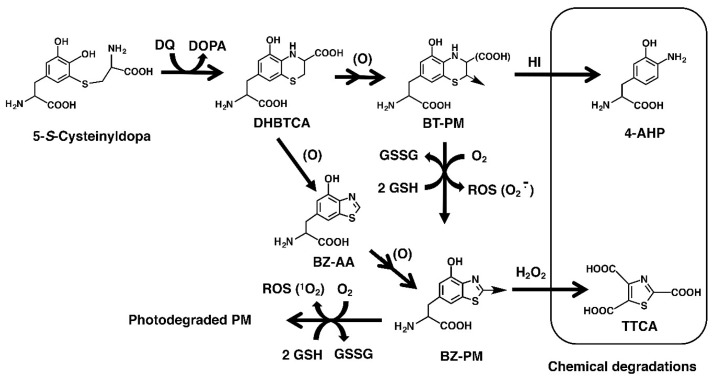
The generation of benzothiazine (BT) and benzothiazole (BZ) moieties of PM via oxidation of 5*S*CD. Tyrosinase-catalyzed oxidation of L-tyrosine in the presence of L-cysteine yields cysteinyldopa isomers [23,24,25,26,27]. 5*S*CD is converted to DHBTCA (7-(2-Amino-2-carboxyethyl)-5-hydroxy-dihydro-1,4-benzothiazine-3-carboxylic acid) through a redox reaction with dopaquinone (DQ) and then to the BT moiety (BT-PM) during the production of PM. DHBTCA also undergoes ring contraction giving BZ-AA (6-(2-amino-2-carboxyethyl)-4-hydroxybenzothiazole). BZ-AA is oxidized to form the BZ moiety (BZ-PM). UVA promotes the oxidative conversion of BT-PM to BZ-PM and finally to photodegraded PM, and during these processes, ROS, such as superoxide anions and singlet oxygen, are generated [37]. The present study shows the depletion of GSH and the production of H_2_O_2_ during these processes. The BT moiety is estimated by the quantification of 4-amino-3-hydroxyphenylalanine (4-AHP) by hydroiodic acid (HI) hydrolysis while the BZ moiety is estimated by the quantification of thiazole-2,4,5-tricarboxylic acid (TTCA) produced by alkaline H_2_O_2_ oxidation.

It is crucially important to determine whether BZ-PM is photoreactive because PM in human skin has recently been shown to consist predominantly of BZ moieties [35]. When we consider the pathogenesis of human melanoma, it is also important to examine whether enhancement of the pro-oxidant activity of PM depends on its structural moieties being BT-PM and/or BZ-PM. It has been recently shown that PM has the capacity to promote pro-oxidant activity even in the dark, although the type of PM was not examined [28]. In addition, the biological effects of UVA are now drawing much attention because of the recent finding that cyclobutane pyrimidine dimers are generated in melanocytes for >3 h after exposure to UVA (“dark CPDs”), a process that involves UV-induced reactive oxygen and nitrogen species and melanin pigment [11]. Considering that it is difficult for humans to live without being exposed to sunlight, we need to know exactly which moiety in PM enhances the pro-oxidant activity of PM by UVA (Figure 2).

The main intracellular storage pool of CySH is glutathione (GSH), which is also the most important cellular antioxidant substance. Thus, PM synthesis may deplete GSH stores and sensitize melanocytes to oxidative damage eventually resulting in carcinogenesis [38]. In this study, we used a physiological dose of UVA (3.5 mW/cm^2^), which is similar to the irradiance in Greece during the midday of June [39], and we compared the enhancement of pro-oxidant activity using synthetic PM with different ratios of BT and BZ moieties and with yellow, black, and albino mouse hairs. The results show that BZ-PM is more potent than BT-PM in promoting GSH depletion and various types of ROS generation when irradiated with UVA. We then compared the UVA-induced pro-oxidant activity of 7-(2-amino-2-carboxyethyl)-5-hydroxy-1,4-benzothiazine-3-carboxylic acid (DHBTCA) and 6-(2-amino-2-carboxyethyl)-4-hydryxybenzothiazole (BZ-AA), monomeric BT and BZ moieties, respectively [29]. Finally, we examined the effects of ROS scavengers to see which ROS participate in the pro-oxidant activity. The results show that UVA enhances the pro-oxidant activity of PM, in particular BZ-PM.

## 2. Results

### 2.1. Characterization of the Experimental Model of Native (Untreated) PM, Heated PM, and Irradiated PM

Firstly, we prepared synthetic PM from a mixture of 3,4-dihydroxyphenylalanine (DOPA) and CySH (1:1 molar ratio) using mushroom tyrosinase, then subjected it to structural alteration by heating or by UVA irradiation [26] to prepare PM mimicking human epidermal melanin. Del Bino et al. [35] showed that epidermal melanin from very light to tan human skin consists of approximately 70% BZ moiety and 30% BT moiety. We analyzed the native PM, heated PM, and UVA-irradiated PM for BT-PM and BZ-PM (Table 1). The BT and BZ moieties in PM were determined using our methods of hydroiodic acid (HI) hydrolysis and alkaline hydrogen peroxide (H_2_O_2_) oxidation, respectively (Figure 2) [40,41,42]. BT and BZ moieties were calculated from the values of 4-amino-3-hydroxyphenylalanine (4-AHP) and thiazole-2,4,5-tricarboxylic acid (TTCA), which are specific degradative markers [41,42,43,44]. The native PM was found to consist of 76% BT moiety. When this native PM was heated in solution at 100 °C for 6 days, its structure was changed markedly and consisted of 97% BZ moiety. On the other hand, when the native PM was UVA irradiated at 3.5 mW/cm^2^ for 6 days, the irradiated PM consisted of 60% BZ moiety.

We also measured UV-visible spectra of three synthetic PMs. As shown in Figure S1, the heated PM and the irradiated PM had 1.48 and 1.56 times greater absorbances compared to the native PM in the UVA region (320–400 nm), respectively [34].

### 2.2. Depletion of Glutathione and Production of H_2_O_2_ from Synthetic PMs

**Figure 3:**
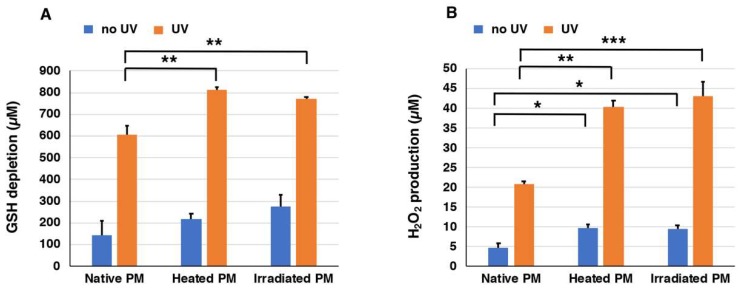
The depletion of GSH (**A**) and the production of H_2_O_2_ (**B**) by synthetic PMs. One thousand micromolar (1000 µM) GSH was added to the three synthetic PMs and UVA irradiation was carried out for 7 h. The data are means ± standard error of the mean (SEM) from three experiments. There were statistically significant differences (* *p* < 0.05, ** *p* < 0.01, *** *p* < 0.001).

We prepared three types of synthetic PMs, native PM, heated PM, and UVA-irradiated PM, that differed in their ratios of BT-PM to BZ-PM. In order to examine the UVA-induced pro-oxidant activities of these synthetic PMs (40 µg/mL), they were exposed to UVA in the presence of 1000 µM GSH, and the depletion of GSH and the generation of H_2_O_2_ were measured. This concentration of GSH is a physiological one, as GSH concentrations in cells range from 0.5 to 10 mM [45,46]. We followed the time course of the decrease of GSH and the increase of H_2_O_2_ under UVA-irradiated and non-irradiated conditions for up to 7 h. Figure S2A shows the time course of GSH depletion in solutions of the three synthetic PMs with or without exposure to UVA. GSH levels in heated PM, UVA-irradiated PM, and native PM decreased rapidly and almost linearly as the time after UVA irradiation passed. On the other hand, GSH depletion proceeded much slower in the absence of UV irradiation. The effect of exposure to UVA was compared at 7 h of irradiation. Figure 3A shows that the decrease of GSH was promoted dramatically by UVA irradiation. The depletion of 606 µM, 811 µM, and 773 µM GSH was observed for native PM, heated PM, and UVA-irradiated PM, respectively, during the 7 h of irradiation. On the other hand, the depletion of 145 µM, 218 µM, and 275 µM GSH in the controls without UVA irradiation was observed for native PM, heated PM, and UVA-irradiated PM, respectively. This indicated that the depletion of GSH by UVA irradiation increased 2.8–4.2 times compared to no UVA irradiation. Heated PM and UVA-irradiated PM promoted GSH depletion significantly (*p* < 0.01) greater than native PM. These results indicated that the BZ moiety promotes the oxidation of GSH by UVA more than the BT moiety.

Figure S2B shows the time course of H_2_O_2_ production in solutions of synthetic PMs exposed to UVA. The production of H_2_O_2_ during 7 h of UVA irradiation of heated PM, UVA-irradiated PM, and native PM increased almost linearly as the UVA irradiation time passed. On the other hand, H_2_O_2_ production did not increase in the case of no UV irradiation. The effect of UVA was compared at 7 h of irradiation. Figure 3B shows the production of H_2_O_2_ by UVA irradiation after the addition of 1000 µM GSH to synthetic PMs. Twenty-one micromolar (21 µM), 40 µM, and 43 µM H_2_O_2_ were measured in native PM, heated PM, and UVA-irradiated PM, respectively. This indicated that the production of H_2_O_2_ by UVA irradiation increased 4.0–4.3 times with UVA irradiation compared to no UVA irradiation. Heated PM and UVA-irradiated PM promoted the production of H_2_O_2_ significantly (*p* < 0.01) greater than the native PM both in the no UV and in the UV groups. Again, these results indicated that the BZ moiety promotes the production of H_2_O_2_ by UVA more than the BT moiety.

### 2.3. Depletion of Glutathione and Production of H_2_O_2_ from Natural Hair Melanins

Subsequently, we extended the experiments on synthetic PMs to natural melanins to confirm that UVA indeed enhances the pro-oxidant activity of natural PM. As typical natural melanins, we used hairs from C57BL recessive yellow (*e*/*e*), black (*a*/*a*), and albino (*c*/*c*) congenic mice as models of PM, EM, and no melanin, respectively. We used homogenates of hairs instead of isolated melanins to avoid undesirable structural modifications that occur during the isolation procedure [47]. Table 2 shows the melanin contents of the hairs of mice used in these experiments. The ratios of PM to EM in the recessive yellow and black mice were 4.4 and 1.4 × 10^−3^, respectively. PM in mouse hair is known to consist mainly of BT moieties [40]. On the other hand, albino mice contain no or negligible amounts of melanin, and thus albino mice can be used as a negative control.

**Figure 4:**
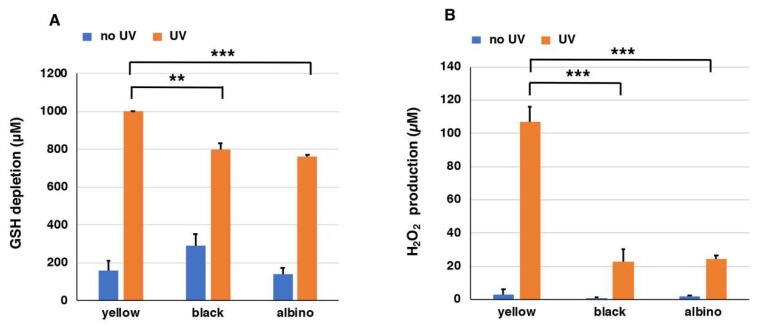
The depletion of GSH (**A**) and the production of H_2_O_2_ (**B**) by mouse hair homogenates. One thousand micromolar (1000 µM) GSH was added to homogenates of yellow, black, and albino mouse hairs and UVA irradiation was carried out for 24 h. The data are means ± SEM from three experiments (** *p* < 0.01, *** *p* < 0.001).

Figure 4A shows that homogenates of yellow mouse hairs consumed GSH completely during the 24 h of UVA irradiation. On the other hand, 802 µM and 762 µM GSH were consumed in homogenates of hairs of black and albino mice, respectively. The depletion of GSH without UVA irradiation proceeded only slowly with depletion less than 300 µM in the three PMs. The rate of GSH depletion in homogenates of yellow hairs was enhanced by UVA irradiation by >6.3-fold.

Using homogenates (1 mg/mL) of yellow, black, and albino mouse hairs, we examined the depletion of GSH (1000 µM) as we did for the synthetic PMs. We followed the time course of the decrease of GSH and the increase of H_2_O_2_ under UVA-irradiated and non-irradiated conditions for up to 24 h. Figure S3A shows the time course of GSH depletion in suspensions of mouse hairs exposed to UVA. GSH levels in yellow, black, and albino mouse hairs decreased rapidly and almost linearly as the time of UVA irradiation passed. The effect of UVA was compared at 24 h of irradiation.

We also examined the production of H_2_O_2_ from mouse hairs by UVA irradiation after the addition of 1000 µM GSH. Figure S3B shows the time course of H_2_O_2_ production in suspensions of mouse hairs exposed to UVA. In suspensions of yellow mouse hairs during the 24 h of UVA irradiation, H_2_O_2_ was produced at a high level as the time of UVA irradiation passed, while it remained at much lower levels in suspensions of black and albino hairs. On the other hand, the amount of H_2_O_2_ did not increase in the case of no UV irradiation. The effect of UVA was compared at 24 h of irradiation. Figure 4B shows that a suspension of yellow mouse hair produced 107 µM H_2_O_2_ during 24 h of UVA irradiation, while it produced only 3 µM H_2_O_2_ without UVA irradiation. On the other hand, suspensions of black and albino mouse hairs produced 23 µM and 25 µM H_2_O_2_ during 24 h of UVA irradiation, respectively. Suspensions of black and albino mouse hairs without UVA irradiation did not produce H_2_O_2_ (<2 µM). The suspension of yellow mouse hairs promoted the production of H_2_O_2_ significantly more than the suspensions of black or albino mouse hairs with UVA irradiation. The rate of H_2_O_2_ production in the suspension of yellow mouse hairs was enhanced 31-fold by UVA irradiation.

**Figure 5:**
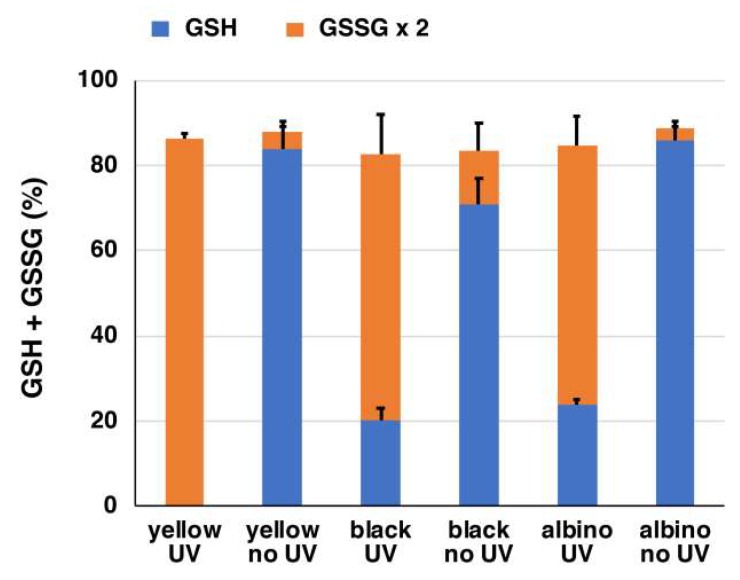
The ratio of GSH and oxidized glutathione (GSSG) in mouse hair homogenates with or without UVA irradiation. The data are means ± SEM from three experiments.

We also analyzed oxidized glutathione (GSSG) at the same time to examine the fate of reduced GSH after depletion (Figure 5). Most (>80%) of the GSH depletion was accounted for by the oxidation to GSSG following UVA irradiation.

### 2.4. Depletion of Glutathione and Production of H_2_O_2_ from DHBTCA or BZ-AA and the Effects of ROS Scavengers

**Figure 6:**
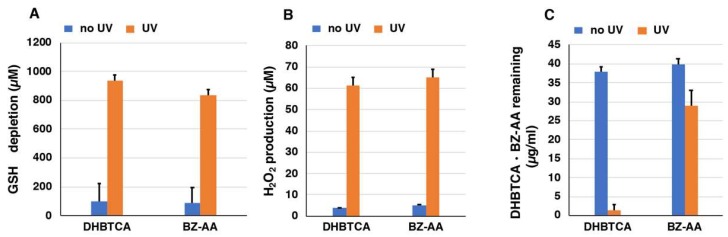
The depletion of GSH (**A**) and the production of H_2_O_2_ (**B**) by DHBTCA and BZ-AA and their disappearance (**C**). One thousand micromolar (1000 µM) GSH was added to the DHBTCA or BZ-AA and UVA irradiation was carried out for 7 h. The data are means ± SEM from three experiments.

**Figure 7:**
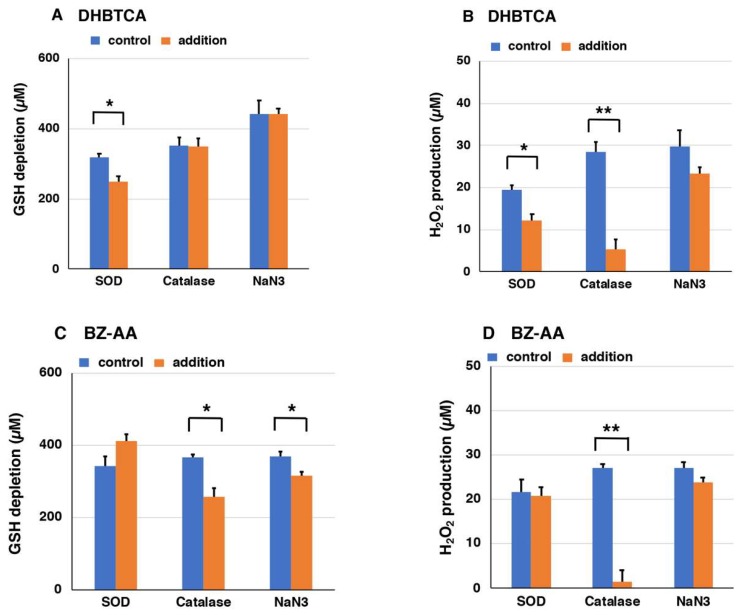
The depletion of GSH (**A**,**C**) and the production of H_2_O_2_ (**B**,**D**) during UVA irradiation of DHBTCA (**A**,**B**) and BZ-AA (**C**,**D**) in the presence of ROS scavengers**.** The data are means ± SEM from three experiments (* *p* < 0.05, ** *p* < 0.01).

Next, to examine the mechanism of the pro-oxidant activity of BT-PM and BZ-PM, we examined how the corresponding monomers behave after UVA irradiation. DHBTCA is a major, BT-type precursor in PM production, while BZ-AA is a BZ-type precursor that is produced following DHBTCA (Figure 2) [26]. Figure S4A,B show that in both DHBTCA and BZ-AA (40 µg/mL), the depletion of GSH and the production of H_2_O_2_ proceeded almost linearly during 7 h of irradiation, although those monomers did not show much difference. On the other hand, both the depletion of GSH and the production of H_2_O_2_ were negligible in the non-irradiated controls. These results were compared for values at 7 h, indicating strong enhancing effects of UVA both in the GSH depletion and in the H_2_O_2_ production (Figure 6A,B). Figure S4C shows the time course of the disappearance of DHBTCA and BZ-AA during the UVA irradiation. DHBTCA was consumed linearly to almost zero with time while BZ-AA was little consumed. These results, shown for the 7 h values (Figure 6C), indicate that BZ-AA, a monomeric BZ moiety, recycles during UVA irradiation to oxidize GSH and produce H_2_O_2_ (or reduce molecular oxygen) but DHBTCA, a monomeric BT moiety, was oxidized during the 7 h irradiation. We then examined the effects of ROS scavengers on DHBTCA and BZ-AA by comparing their pro-oxidant activities. Superoxide dismutase (SOD) dismutates superoxide radicals to H_2_O_2_ and O_2_, while catalase decomposes H_2_O_2_ to O_2_ and H_2_O. NaN_3_ also efficiently scavenges singlet oxygen [48]. Figure 7A,C show GSH depletion during 3 h of UVA irradiation of DHBTCA and BZ-AA solutions in the absence or presence of various ROS scavengers. The production of H_2_O_2_ under the same conditions is shown in Figure 7B,D.

In the irradiated DHBTCA solution, SOD (50 µg/mL) suppressed GSH depletion by 21%, while catalase (50 µg/mL) and NaN_3_ (10 mM) had no effect (Figure 7). In the irradiated BZ-AA solution, catalase and NaN_3_ suppressed GSH depletion by 30% and 15%, respectively, while SOD showed a weak accelerating effect. It should be stressed that although those suppressing effects were limited, they are nevertheless statistically significant from the controls carried out simultaneously. In the irradiated DHBTCA solution, SOD and catalase suppressed H_2_O_2_ production by 37% and 82%, respectively, while NaN_3_ had little effect. In the irradiated BZ-AA solution, catalase and NaN_3_ suppressed H_2_O_2_ production by 93%, while SOD and NaN_3_ had little effect.

## 3. Discussion

Both types of melanin pigment, EM and PM, are redox-active and undergo rapid and repeated redox cycles between the oxidized and reduced states [49]. We recently examined the photoreactivity of synthetic EMs formed by the auto-oxidation of DOPA or by the enzymatic oxidation of 5,6-dihydroxyindole-2-carboxylic acid (DHICA) as well as synthetic PMs obtained by the enzymatic oxidation of 5-*S*-cysteinyldopa or a 1:1 mixture of DOPA and CySH [49]. Superoxide anions and singlet oxygen were photogenerated by these synthetic melanins, albeit with different efficiencies. These results showed that in the short wavelength part of UVA (320–360 nm), the photogeneration efficiency of singlet oxygen is higher for synthetic pheomelanins than for DHICA-melanin and DOPA-melanin. We considered that the excitation of different melanin chromophores is responsible for the photogeneration of singlet oxygen in the wavelength range. From this result, we conclude that each subunit of melanin plays an important role in determining which species of ROS is efficiently produced.

The mechanism of PM cytotoxicity is now thought to occur as follows: When the dihydrobenzothiazine (DHBT) moiety in PM is oxidized to the quinone-imine form [13], superoxide anions are generated from molecular oxygen. The superoxide anions can react rapidly with antioxidants, such as GSH or CySH [37], and this scavenging of superoxide anions leads to the production of H_2_O_2_. It is known that superoxide anions are generated from melanin, which is accelerated by UVA irradiation [5,10,34,35,49]. The depletion of antioxidants, such as GSH and nicotinamide adenine dinucleotide (phosphate) (NAD(P)H), results in the accumulation of endogenous ROS, which may increase the vulnerability of cells [31]. Thus, the production of ROS due to UVA irradiation of PM directly damages DNA, and the depletion of GSH, NAD(P)H, and other cellular antioxidants leads indirectly to DNA damage (Figure 1). As a result, these interactions may eventually lead to carcinogenic effects [38]. The orthoquinone-imine form of PM is highly reactive and is reduced back to the DHBT form or is gradually and irreversibly converted to the BZ form during heating or UV irradiation [26,35,50]. It has been generally thought that the BZ moiety is not related to the redox reaction of PM because of the chemical stability of the BZ structure compared to the BT/DHBT structure [13]. A direct H-atom transfer from the thiol to free radical moieties of the pigment seems to also proceed through the interaction of GSH with pheomelanin. Reduced PM would be reoxidized by oxygen, ROS, and restoring the free-radical [13].

In the present study, we investigated whether the pro-oxidant activity of PM is enhanced by UVA and further investigated whether BT-PM and/or BZ-PM are involved in the production of different species of ROS. The UVA radiance of 3.5 mW/cm^2^ was used in this study as well as in past studies [10,35,51]. This study showed that UVA irradiation promotes the pro-oxidant activity of synthetic PMs, pheomelanic mouse hair, and the monomeric structural moieties of PM by increasing both the depletion of GSH and the production of H_2_O_2_ compared to the non-irradiated controls.

In synthetic PMs, after 7 h of irradiation, the depletion of GSH was 3–4 times faster compared to each non-irradiated control. H_2_O_2_ was also generated about 4 times faster. As for the mechanism of the pro-oxidant activity of PM, Napolitano et al. [13] proposed that the oxidation of thiols by the BT moiety of PM that yields disulfides is accompanied by the reduction of BT to the DHBT moiety [13,29], and this reaction proceeds with a one-electron transfer from the thiol to the BT moiety to produce thiyl radicals and melanin radicals. Two thiyl radicals combine to form a disulfide and two melanin radicals exchange electrons to form the DHBT and BT moieties. It has been known for some years that superoxide anions are generated from melanins and that this process is accelerated by UV radiation [5,32]. In addition, our recent studies have confirmed that this type of redox reaction that produces superoxide anions is accelerated by UVA irradiation in synthetic EM and PM [10,50]. In those studies, it was also shown that singlet oxygen is generated along with superoxide anions from synthetic melanins. More recently, we also showed that synthetic DOPA-PM (PM from DOPA plus CySH) oxidizes GSH, CySH, ascorbic acid, and NADH with a concomitant production of ROS more effectively than DOPA-EM [34]. Among the superoxide anions generated, some dismutate to form H_2_O_2_ and molecular oxygen (self-mutation). The remaining superoxide anions are able to react rapidly with antioxidants, such as thiols [37] and ascorbic acid [52].

Recent data reported that PM act as potent photosensitizers leading to production of toxic ROS [15,53]. Panzella et al. characterized how PM isolated from human red hair impacts the cellular redox system, and that GSH and NADH were both significantly diminished by PM [29]. The study [35] showing that PM in human skin has mainly BZ moieties, not BT moieties, highlighted an important issue in the rising incidence of sun-related skin diseases, particularly melanoma. As PM is known to exert cytotoxicity to melanocytes through the production of ROS [38], an important goal of our study was to understand the chemical nature of the chromophore responsible for the photogeneration of ROS by the photo-irradiated PM.

The heated PM and the irradiated PM had about 1.5 times greater absorbances compared to the native PM in the UVA region (320–400 nm). This is consistent with the greater photoreactivity of the heated PM and the irradiated PM observed in this study. Our results showed that heated PM with a 97% BZ structure promotes GSH depletion by 1.3 times and H_2_O_2_ production by 2.0 times compared to native PM with a 76% BT structure. This indicates that heated PM containing mostly BZ moieties enhances pro-oxidant activity by UVA more than native PM containing mainly BT moieties. Irradiated PM with a 60% BZ structure also showed a similar increase in pro-oxidant activity compared to native PM. These trends were also observed for non-irradiated PMs in H_2_O_2_ production, although statistically not significant in GSH depletion. These results suggest that BZ-PM possesses a higher pro-oxidant activity than BT-PM, which has not hitherto been described. It is generally believed that the BT structure is responsible for the redox reaction of PM [13,29,54]. However, our results that BZ-PM is more potent than BT-PM in promoting GSH depletion and ROS generation when irradiated with UVA are consistent with a recent study by Zadlo et al. [55]. They analyzed the efficiency of DHBTCA and BZ-AA, two main constituents of PM, to photogenerate and quench singlet oxygen when excited at 320 nm. BZ-AA generated singlet oxygen with a quantum yield very close to that of fluorescein, an effective singlet oxygen generator. On the other hand, the efficacy of DHBTCA to photogenerate singlet oxygen under similar conditions was at least 5-fold lower than BZ-AA.

When homogenates of yellow mouse hairs were irradiated with UVA for 24 h, the depletion of GSH was enhanced by >6.3 times and the production of H_2_O_2_ was increased by 35 times compared with the non-irradiated control. There was not much apparent difference in GSH depletion between yellow and black hairs. However, considering that the PM content of yellow mouse hairs is 14.5 µg/mg hair, while the EM content of black mouse hairs is 58.6 µg/mg hair, the effect of PM on pro-oxidant activity appears to be much more remarkable. One limitation of this study is the high background value from albino hairs (Figure 4A,B), which have non-melanin components that are present in melanosomes, such as proteins, lipids, carbohydrates, and metal ions. Those components could affect the observable photo-reactivity. For example, proteins in the eye are known to be important targets of photochemical damage. The photoionization of tyrosine and tryptophan residues, under the effects of UV radiation, leads to aromatic free radicals. It is known that *N*-formylkynurenine, an important tryptophan photoproduct, acts as an endogenous photosensitizer to generate singlet oxygen and superoxide anions [55,56]. Nevertheless, it should be emphasized that when exposed to UVA, H_2_O_2_ production from yellow mouse hairs was ca. 5 times greater than from black or albino mouse hairs.

Reports suggesting that the pro-oxidant activity of PM does not necessarily require UVA radiation have increased in recent years. It has been known that the presence of *MC1R* gene variants results in a higher melanoma risk, which is independent of skin type and hair color [57,58,59]. In a recent large-scale epidemiological study, Wendt et al. reported that the risk of melanoma in MC1R variants (redheads) increases irrespective of sun exposure [60]. Their study showed that individuals carrying two MC1R variants were at a higher melanoma risk independent of UV-exposure symptoms, compared to subjects with wild-type MC1R [61]. Morgan et al. [38] proposed two concepts by which PM might consume major antioxidants and/or increase ROS generation directly: (i) PM might generate ROS that cause oxidative DNA damage and lipid peroxidation, and/or (ii) PM might consume cellular antioxidant stores and make cells more vulnerable to elevated ROS levels [13,29]. Nevertheless, it should be stressed that the majority of Caucasians are not MC1R gene variants [59] and thus it is possible that their susceptibility to PM cytotoxicity may be exacerbated by UVA radiation.

On the higher-energy end of the electromagnetic spectrum, ionizing nuclear radiation may also interact with PM. For example, among birds exposed to radiation at Chernobyl in the Ukraine, the TTCA (BZ moiety)/4-AHP (BT moiety) ratio in their feathers increases under background radiation with the depletion of GSH levels in pheomelanic birds, suggesting that PM might be toxic when combined with nuclear radiation [62]. It is possible that the synthesis of PM depletes GSH stores, which could make melanocytes more susceptible to oxidative damage and carcinogenesis. The GSH depletion hypothesis has been used to explain other phenomena in animals that have pheomelanic coloring. Galván et al. showed that the PM coat color in wild boars is associated with increased levels of oxidative stress and lower GSH levels in muscle cells [63]. If GSH depletion is to blame for the carcinogenicity of PM, supplementation with antioxidants may prove to be effective in reducing the increased melanoma risk of redheads.

We measured the pro-oxidant activities of DHBTCA and BZ-AA. Both of those PM monomers showed rapid rates of GSH depletion and H_2_O_2_ production only when irradiated with UVA. Subsequently, in order to examine the involvement of ROS, various ROS scavengers were added to DHBTCA and to BZ-AA and their effects were compared. The GSH depletion in DHBTCA was partially suppressed by SOD, while that in BZ-AA was partially suppressed by catalase and by NaN_3_. The H_2_O_2_ production in DHBTCA was also suppressed by SOD. These results suggested that redox reactions may occur via singlet oxygen and H_2_O_2_ in BZ-AA and via superoxide anions in DHBTCA. The suppression of GSH depletion (and H_2_O_2_ production) was limited, although statistically significant. This may be, at least in part, ascribed to the direct (ROS-independent) oxidation of GSH by PM radicals, as proposed by Naples’ group [13,29].

Lembo et al. reported that red hair PM, and to a minor extent black hair EM, works as a direct pro-inflammatory and pro-oxidant agent independently from light exposure in keratinocyte cultures [64]. On the other hand, Lawrence et al. demonstrated that UV/visible boundary wavelengths (385–405 nm) cause significant biologically relevant damage in vitro and in vivo, including dark CPD formation [22]. This damage is possibly caused by oxidative stress generated by chromophores in the skin, such as melanins, protoporphyrin IX, and β-carotene, that absorb strongly in this region.

In conclusion, this is the first study to examine the effects of photoreactivity of PM by UVA irradiation on the pro-oxidant activity of PM and to compare the efficacies of BT-PM and BZ-PM. We showed that UVA enhances the pro-oxidant activity of PM and that BZ-PM is more reactive than BT-PM. The pro-oxidant activity of PM is enhanced 3–4-fold by UVA irradiation compared with no irradiation. This enhancement might be considered to be physiologically insignificant. However, it is possible that the additive effects of the depletion of antioxidants and the generation of ROS would overwhelm the cellular defense mechanism against oxidative stress, resulting in DNA damage and eventually leading to melanoma and keratinocyte carcinoma (Figure 1).

## 4. Materials and Methods

### 4.1. Materials

Tyrosinase (from mushrooms, specific activity 1715 U/mg), horseradish peroxidase, Ampliflu^TM^ Red reagent (1-acetyl-3,7-dihydroxyphenoxazine), L-CySH, SOD (from bovine erythrocytes, specific activity 3000 U/mg), catalase (from bovine liver, 44,000 U/mg), and hexane were purchased from Sigma-Aldrich (St. Louis, MO, USA). Dimethyl sulfoxide, 3,5-di-*tert*-butyl-1,2-benzoquinone (DBBQ), 2-mercaptoethanol (thioglycol), GSH, NaN_3_, formic acid, and methanol (HPLC (High performance liquid chromatography) grade) were purchased from Wako Pure Chemical Industry (Osaka, Japan). H_2_O_2_ was purchased from Mitsubishi Gas Chemical Company, Ltd. (Tokyo, Japan). Perchloric acid was purchased from Katayama Chemical Industries Co., Ltd. (Osaka, Japan). All other chemicals were of the highest purity commercially available. The highest purity Milli-Q water (Milli-Q Advantage, Merck Millipore Co., Tokyo, Japan) was used throughout this study to avoid contamination with metal ions. Hairs from recessive yellow (*e*/*e*), black (*a*/*a*), and albino (*c*/*c*) mice were obtained from the National Institute of Radiological Sciences, Chiba, Japan. We prepared DHBTCA using a method similar to that reported for the dihydrobenzothiazine derivative of 5*S*CD [65] and BZ-AA as described by Di Donato et al. [66] with some modifications [26].

### 4.2. Instruments

For UV irradiation, we used an Oriel 300W Solar simulator (Oriel Instruments, now the Newport Corporation, Stratford, CT, USA). The dose used was measured using a Photo-Radiometer (Delta Ohm srl, HD 2302.0; Casella di Selvazzano (Pd), Italy) prior to each exposure. For UVA (320–400 nm), UVB wavelengths were removed using a combination of a cold filter, a Longpass filter/UV 325 nm, and a Red reject UV filter/340 nm (Asahi Spectra Co., Tokyo, Japan). Dichromatic mirrors were used to minimize wavelengths above 400 nm. The dose of UVA used was 3.5 mW/cm^2^ (0.04 mW/cm^2^ as UVB), which is similar to the irradiance in Greece during midday in June [39].

For measurements of H_2_O_2_, the maximum absorption wavelength of 571 nm possessed by red fluorescent resorufin in which Ampliflu^TM^ Red was oxidized was used.

UV-visible spectra were measured with a JASCO V-630 UV-VIS spectrophotometer (JASCO Co., Tokyo, Japan).

We used a HPLC system consisting of an analytical UV/VIS detector, a JASCO pump (JASCO Co., Tokyo, Japan), a Shiseido C18 column (Capcell Pak MG; 4.6 × 250 mm; 5 µm particle size, Shiseido, Tokyo, Japan), and a JASCO UV-visible detector (JASCO Co., Tokyo, Japan).

### 4.3. Biochemical Analyses

Synthetic PM (native PM) was prepared by the tyrosinase-catalyzed oxidation of L-DOPA and L-CySH (1:1 molar ratio) according to the method described elsewhere [42]. Heated PM and UVA-irradiated PM were prepared by heating a solution (200 µg/mL in 50 mM sodium phosphate buffer, pH 6.8) at 100 °C and UVA irradiation of native PM for 6 days, respectively. Synthetic PMs (native PM, heated PM, and UVA-irradiated PM) were diluted to a concentration of 40 µg/mL in the presence of 1000 µM GSH in 50 mM sodium phosphate buffer (pH 6.8). As controls, synthetic PMs without UVA irradiation were also prepared at the same time. After UVA irradiation for 0, 1, 2, 4, 7, and 24 h, aliquots of the mixtures were withdrawn for the assays of GSH and H_2_O_2_.

DHBTCA and BZ-AA were diluted to a concentration of 40 µg/mL in 50 mM sodium phosphate buffer (pH 6.8) containing 1000 µM GSH and each ROS scavenger. As a control, mixtures without UVA irradiation were also prepared at the same time. After UVA irradiation for 0 or 3 h, aliquots of the mixtures were withdrawn for the assays of GSH and H_2_O_2_. Catalase (hydrogen peroxide degrading enzyme), sodium azide (singlet oxygen scavenger), and superoxide dismutase (SOD (superoxide anion degrading enzyme)) were used as ROS scavengers. Experimental variables for them were as follows: SOD (50 µg/mL), catalase (50 µg/mL), and NaN_3_ (10 mM).

Hairs from recessive yellow (*e*/*e*), black (*a*/*a*), and albino (*c*/*c*) mice were homogenized in water at 10 mg/mL using a Wheaton Ten-Broeck tissue grinder and were diluted to a concentration of 1 mg/mL in the presence of 1000 µM GSH containing 50 mM sodium phosphate buffer (pH 6.8). As a control, hairs without UVA irradiation were also prepared at the same time. After UVA irradiation for 0, 3, 7, and 24 h, aliquots of the mixtures were withdrawn for the assays of GSH and H_2_O_2_.

Levels of GSH in the oxidation mixtures were analyzed using an HPLC method [67]. For synthetic PMs, we used 40 µL of each oxidation mixture followed by dilution with 360 µL 0.4 M HClO_4_. For mouse hairs, we used 20 µL of each oxidation mixture followed by dilution with 180 µL 0.4 M HClO_4_. Fifty microliters taken from each 200 µL mixture were again diluted with 50 µL 0.4 M HClO_4_. One hundred microliters of each mixture were mixed with a freshly prepared ethanol solution of 100 µL 1 mM DBBQ and were shaken for 30 min at 30 °C.

To measure levels of GSSG, 20 µL 5 mM 2-mercaptoethanol were added to each 20 µL oxidation mixture and mixed. Twenty microliters (20 µL) of 0.4 M Na_2_CO_3_ were then added and the mixture was shaken for 30 min at 30 °C. Next, 40 µL 1.2 M HClO_4_ were added and reacted with an ethanol solution of 100 µL 1 mM DBBQ in the same manner as GSH.

Standard solutions each containing 50 µM GSH and CySH (100 µL) or 25 µM GSSG and CySSCy (100 µL) were similarly treated. The HPLC system was modified from the original conditions as follows: a mobile phase of 0.4 M HCOOH: methanol, 30:70 (*v*/*v*) was used with a UV detector at 294 nm and a column temperature of 45 °C. GS-DBBQ and CyS-DBBQ adducts eluted at 10.4 and 12.0 min, respectively.

H_2_O_2_ was analyzed spectrophotometrically after reaction with the chromogen Ampliflu^TM^ Red to form a red pigment having an absorption maximum at 568 nm [48] closely following the manufacturer’s instructions (Invitrogen, Tokyo, Japan). Briefly, the chromogen solution was prepared by adding 50 µL Ampliflu^TM^ Red solution (1.54 mg in 0.6 mL DMSO) and 100 µL horseradish peroxidase (100 U/mL) to 4.85 mL 50 mM sodium phosphate buffer, pH 7.4. A sample solution properly diluted with the buffer (as a standard solution, H_2_O_2_ was diluted with buffer solution to a final concentration of 20 µM) was mixed with the chromogen solution (200 µL) and the mixture was left at room temperature for 10 min. Absorption spectra were then measured between 450 and 650 nm. Backgrounds for the reagent oxidized and melanin were subtracted using absorption spectra.

Alkaline H_2_O_2_ oxidation to measure PTCA was performed as described in Ito et al. [41]. HI reductive hydrolysis to measure 4-AHP was performed as described in Wakamatsu et al. [42]. Soluene-350 solubilization to measure TM was performed as described elsewhere with a minor modification [41].

### 4.4. Statistical Analyses

Students’ *t*-test for paired samples (one-tailed) was employed with Microsoft Excel software (Microsoft Corp., Microsoft^®^Excel^®^2013, Redmond, WA, USA).

## Figures and Tables

**Table 1:** The ratio of BT-PM (Benzothiazine pheomelanin) and BZ-PM (Benzothiazole pheomelanin) in synthetic PMs (pheomelanin).

Synthetic PM	4-AHP (µg/mg)	TTCA (µg/mg)	BT-PM (µg/mg)	BZ-PM (µg/mg)	BZ/(BZ + BT) × 100 (%)
Native PM	149.6	9.5	1047.2	323.0	24
Heated PM	1.9	12.6	13.3	428.4	97
Irradiated PM	44.9	13.9	314.3	472.6	60

Levels of BT-PM and BZ-PM were calculated by multiplying 4-AHP (4-Amino-3-hydroxyphenylalanine) and TTCA (Thiazole-2,4,5-tricarboxylic acid) values by the conversion factors 7 and 34, respectively [35]. The data are averages from two separate determinations.

**Table 2:** The contents of total melanin (TM), eumelanin (EM), PM and the PM/EM ratio in hairs of recessive yellow (*e*/*e*), black (*a*/*a*), and albino (*c*/*c*) mice.

Coat Color Phenotype	TM (µg/mg)	EM (µg/mg)	PM (µg/mg)	PM/EM Ratio
Recessive yellow (*e*/*e*)	12.7 ± 1.9	3.3 ± 0.4	14.5 ± 1.8	4.4
Black (*a*/*a*)	50.8 ± 4.6	58.6 ± 4.0	0.08 ± 0.01	1.4 × 10^−3^
Albino (*c*/*c*)	0.0	<0.05	<0.07	-

The data for recessive yellow and black mice are averages ± SEM from three mice each and the values for the albino mice are means of duplicate measurements [46,47].

## Supplementary Materials

Supplementary materials can be found at http://www.mdpi.com/1422-0067/19/10/2889/s1.

## Abbreviations

UV	Ultraviolet
EM	Eumelanin
PM	Pheomelanin
ROS	Reactive oxygen species
KC	Keratinocyte carcinoma
DNA-PF	DNA protection factor
BT	Benzothiazine
BZ	Benzothiazole
BZ-PM	Benzothiazole pheomelanin
GSH	Glutathione
H_2_O_2_	Hydrogen peroxide
BT-PM	Benzothiazine pheomelanin
DHBTCA	7-(2-Amino-2-carboxyethyl)-5-hydroxy-dihydro-1,4-benzothiazine-3-carboxylic acid
BZ-AA	6-(2-Amino-2-carboxyethyl)-4-hydroxybenzothiazole
CySH	Cysteine
5*S*CD	5-*S*-cysteinyldopa
2*S*CD	2-*S*-cysteinyldopa
CPD	Cyclobutane pyrimidine dimer
4-AHP	4-Amino-3-hydroxyphenylalanine
HI	Hydroiodic acid
TTCA	Thiazole-2,4,5-tricarboxylic acid
DOPA	3,4-Dihydroxyphenylalanine
GSSG	Oxidized glutathione
SOD	Superoxide dismutase
DHICA	5,6-Dihydroxyindole-2-carboxylic acid
DHBT	Dihydrobenzothiazine
NAD(P)H	Nicotinamide adenine dinucleotide (phosphate)
MC1R	Melanocortin-1 receptor
DBBQ	3,5-Di-*tert*-butyl-1,2-benzoquinone
HPLC	High performance liquid chromatography
