# Supplementary material for: The Pro-Oxidant Activity of Pheomelanin is Significantly Enhanced by UVA Irradiation: Benzothiazole Moieties Are More Reactive than Benzothiazine Moieties
Source: Int J Mol Sci. 2018 Sep 23;19(10):2889. doi: 10.3390/ijms19102889 (PMC6213070; doi:10.3390/ijms19102889)
Supplement: Supplementary file 1 [file ijms-19-02889-s001.pdf]

Supporting Information

The Pro-oxidant Activity of Pheomelanin is Significantly Enhanced by UVA Irradiation: Benzothiazole Moieties are More Reactive than Benzothiazine Moieties

Hitomi Tanaka[1], Yui Yamashita[1], Kana Umezawa[1], Tomohisa Hirobe[2], Shosuke Ito[1], and Kazumasa Wakamatsu[1]

1 Department of Chemistry, Fujita Health University School of Health Sciences, 1-98, Dengakugakubo, Kutsukake-cho, Toyoake, Aichi 470-1192, Japan

2 Laboratory for Cell Culture and Pathology, Shinjuku Skin Clinic. Kawase Building BF1, Shinjuku 3-17-5, Shinjuku-ku, Tokyo 160-0022, Japan

Figure S1

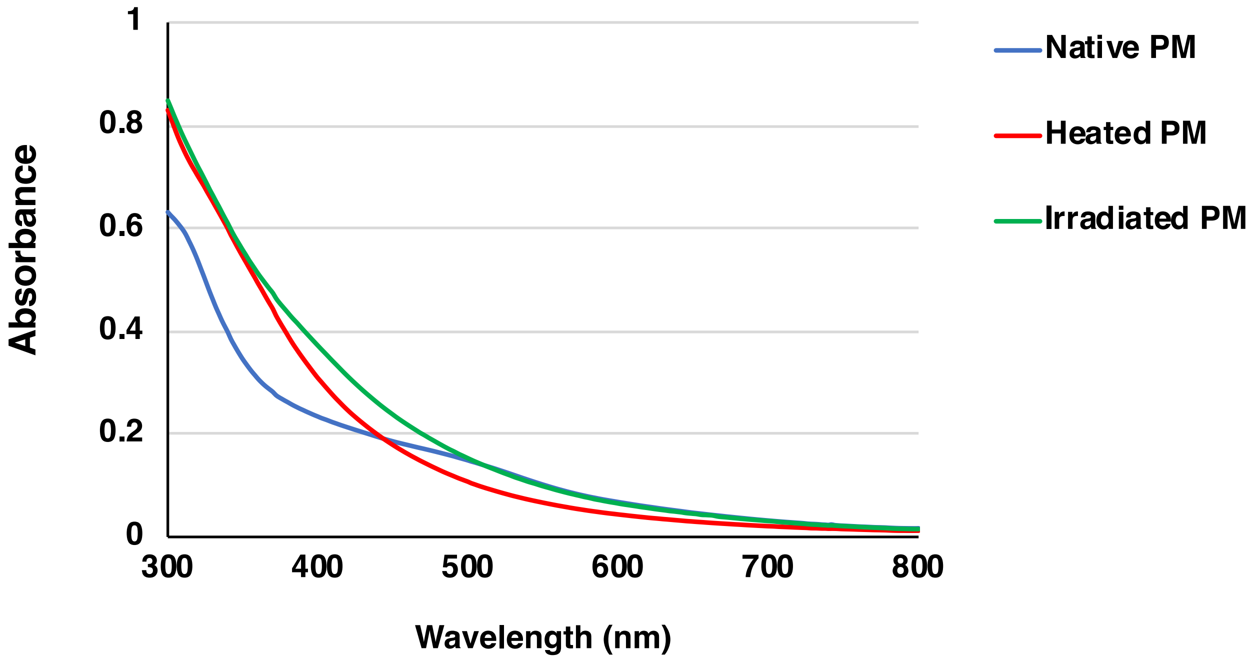

UV-VIS absorption spectra of native PM, heated PM and irradiated PM. Spectra were measured on solutions at a concentration of 40 μg /ml of 0.05 M sodium phosphate buffer, pH 6.8. Spectrophotometric analysis revealed some difference between the three synthetic pheomelanins; native PM had an absorption with a shoulder around 500 nm, but heated PM and irradiated PM had not these shoulders. The irradiated PM and the heated PM showed an increase in integrated absorbance (320-400nm) of 48% and 56% compared to the native PM.

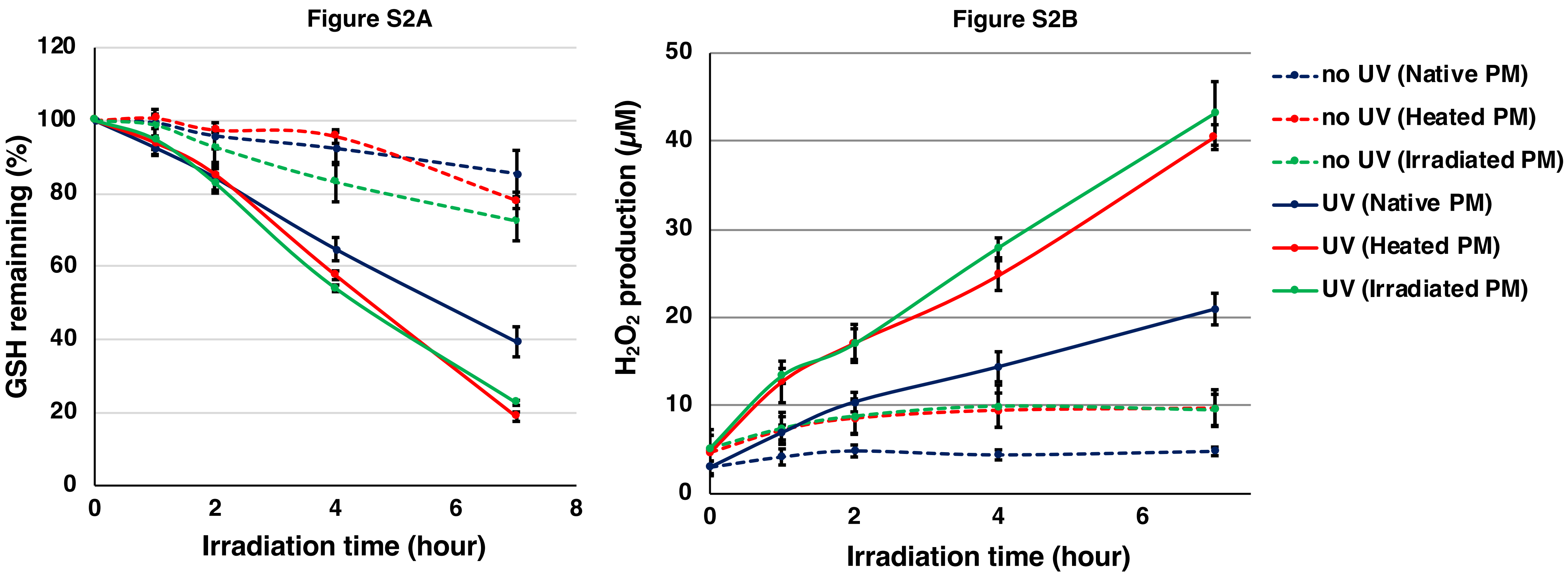

Figure S2A shows the time course of GSH remaining in suspensions of synthetic PMs exposed to UVA. GSH levels in heated PM, UVA-irradiated PM and native PM decreased rapidly after UVA irradiation. After 7 hours of UVA irradiation, 81% and 70% of GSH were depleted in heated PM, UVA-irradiated PM and native PM, respectively. On the other hand, GSH depletion decreased slowly in the case of no UV irradiation. Figure S2B shows the time course of H_2O_2 production in suspensions of synthetic PMs exposed to UVA. H_2O_2 production after 7 hours of UVA irradiation in heated PM, UVA-irradiated PM and native PM increased after UVA irradiation. H_2O_2 production in heated PM and UVA-irradiated PM increased two-fold compared to untreated PM after UVA irradiation. On the other hand, H_2O_2 production did not increase in the case of no UV irradiation. Data reported are means ± SEM of three experiments.

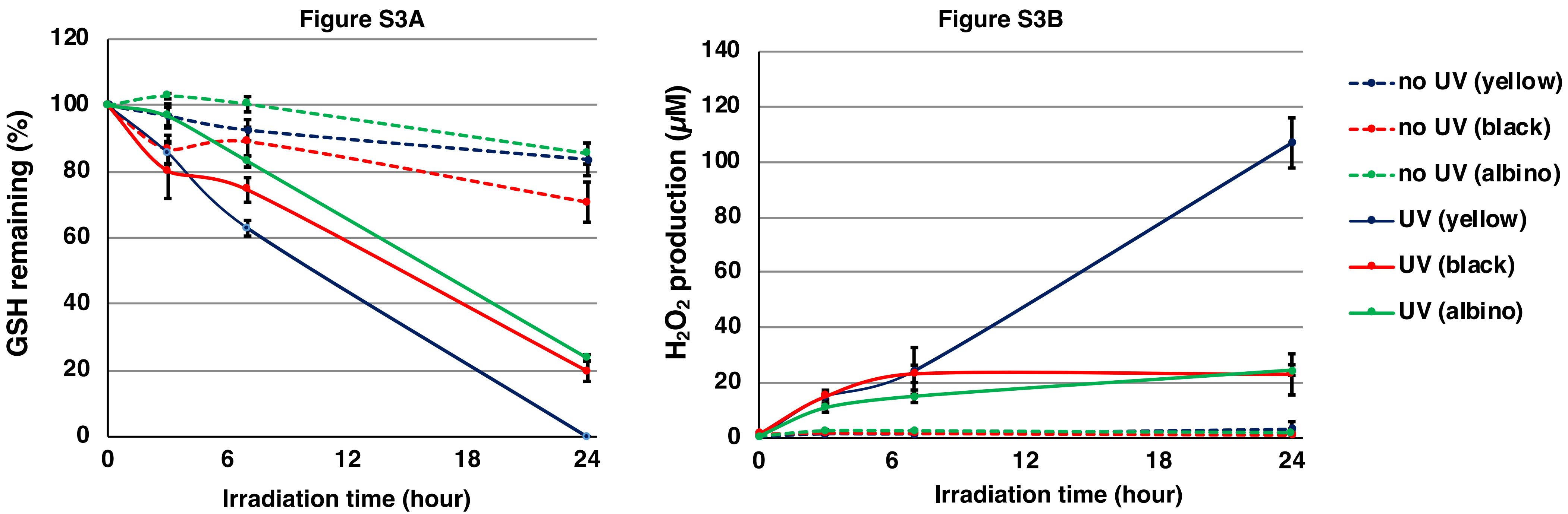

Figure S3A shows the time course of GSH remaining in suspensions of mouse hairs exposed to UVA. GSH in yellow, black and albino mouse hairs decreased rapidly after UVA irradiation. After 24 hours of UVA irradiation, 80% and 75% of GSH were depleted in black and albino hairs, respectively, but GSH was completely depleted in yellow hairs after 24 hours of irradiation. On the other hand, GSH depletion decreased slowly in the case of no UV irradiation. S3B shows the time course of H_2O_2 production in suspensions of mouse hairs exposed to UVA. H_2O_2 in yellow mouse hairs after 24 hours of UVA irradiation was produced at a high level (107 μM) after UVA irradiation, while it was at the trace level in black (23 μM), and albino mouse hairs (25 μM), respectively. On the other hand, H_2O_2 did not increase in the case of no UV irradiation. Data reported are means ± SEM of three experiments.

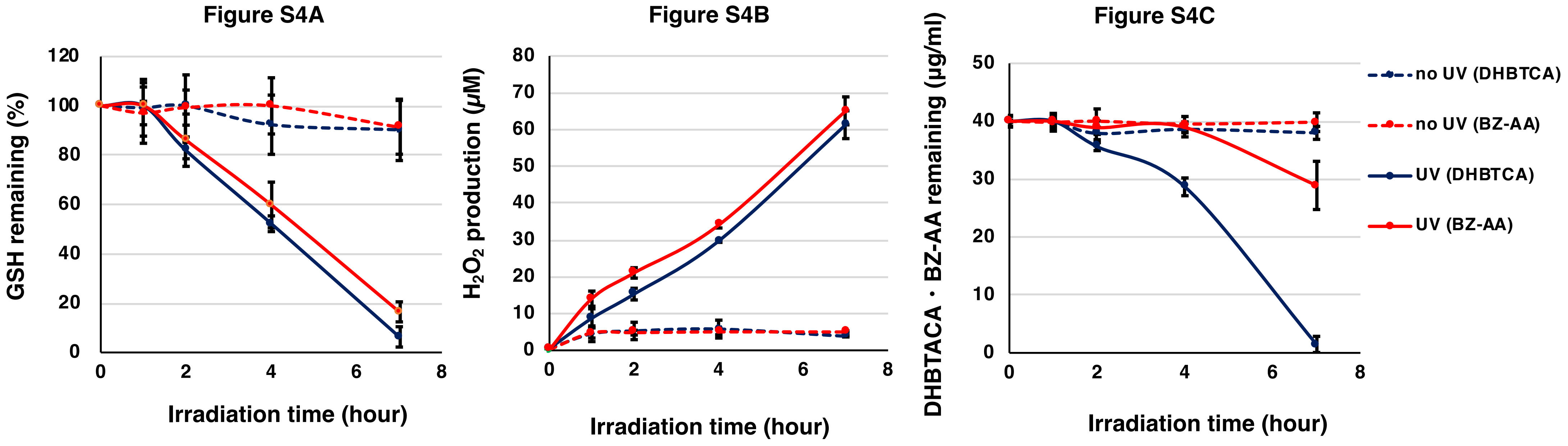

Figure S4A shows the time course of GSH remaining in suspensions of mouse hairs exposed to UVA. GSH in DHBTCA and in BZ-AA decreased rapidly after UVA irradiation. After 7 hours of UVA irradiation, 6% and 17% GSH were remaining in DHBTCA and BZ-AA, respectively. On the other hand, GSH depletion decreased slowly in the case of no UV irradiation. Figure S4B shows the time course of H_2O_2 production in suspensions of DHBTCA or BZ-AA exposed to UVA. H_2O_2 production after 7 hours of UVA irradiation of DHBTCA and BZ-AA increased after UVA irradiation. On the other hand, H_2O_2 production did not increase in the case of no UV irradiation. Figure S4C shows the time course of the remaining DHBTCA and BZ-AA during UVA irradiation. DHBTCA decreased rapidly after UVA irradiation. On the other hand, BZ-AA decreased gradually. After 7 hours of UVA irradiation, the remaining DHBTCA and BZ-AA decreased by up to 2 μg/ml and 29 μg/ml, respectively. However, they did not decrease significantly in the absence of UVA irradiation. Data reported are means ± SEM of three experiments.
